# Empathy Scores and Curriculum Integration at Two Different Levels: A Cross-Sectional Study of Final-Year Medical and Dental Students

**DOI:** 10.7759/cureus.79104

**Published:** 2025-02-16

**Authors:** Amna Faisal, Saira Akhlaq, Naveed Bhatti

**Affiliations:** 1 Community and Preventive Dentistry, Dental College, Heavy Industries Taxila Education City-Institute of Medical Sciences (HITEC-IMS), Taxila, PAK; 2 School of Health Professions Education, Shifa College of Medicine, Shifa Tameer-E-Millat University (STMU), Islamabad, PAK; 3 Orthodontics, Hazrat Bari Imam Sarkar (HBS) Medical and Dental College, Islamabad, PAK

**Keywords:** curriculum design, empathy, harden’s integration ladder, integrated curriculum, medical and dental education

## Abstract

Background: Empathy is a defining trait for healthcare professionals as it fosters trust and understanding between providers and patients. In medical and dental education, empathy is required for enhancing protective health outcomes. Empathy levels vary among medical students throughout their medical schooling and are impacted by factors such as curriculum structure, clinical exposure, and personal characteristics.

Objectives: This study aimed to compare empathy scores corresponding to different levels of curricular integration as defined by Harden, in order to identify any potential differences in empathy levels between two groups.

Methodology: Two study samples, one consisting of medical students and the other of dental students, completed the Kiersma-Chen Empathy Scale-Revised (KCES-R). Empathy scores were assessed across two levels of curriculum integration based on Harden’s integration ladder.

Results: The results indicated that students instructed at a higher level of curricular integration reported higher empathy scores compared to those instructed at a lower integration level.

Conclusion: In conclusion, the findings of this study suggest a positive relationship between curricular integration and empathy scores in medical and dental students.

## Introduction

Empathy and compassion are crucial in healthcare as they enhance patients' comfort, confidence, and self-efficacy, leading to better diagnosis, treatment planning, and adherence to therapy [1]. Compassionate care, which predicts improved patient outcomes, strengthens patient-physician relationships, and increases trust, is a key component of the healthcare mission [2,3]. Empathy can be classified as emotive, cognitive, or a mix of both [4]. Affective empathy involves experiencing, holding, and perceiving other's feelings, while cognitive empathy allows for the comprehension of emotions without the need to take on their feelings [4]. Empathy scores vary between Western and Eastern health professionals, with Eastern countries showing lower scores. Medical students develop empathy skills as they study, but these skills decline due to increased patient interaction and treatment shifts [5-7].

A systematic review revealed a decline in empathy among medical students and residents, which led to decreased satisfaction and burnout [8]. This decrease in empathy can impact patient safety and confidence in healthcare professions [9]. However, empathizing can improve patient satisfaction, reduce malpractice claims, and enhance clinical competence. People-oriented specialties in medical schools tend to have higher empathy scores than technology-oriented specialties [10]. To address this issue, incorporating empathy-enhancing approaches into medical education courses can significantly increase empathy levels. Further, training programs in professionalism and ethics can help preserve empathy levels. Implementing effective courses on empathy can help improve empathy scores among medical practitioners [11,12].

Curricular integration plays a significant role in influencing empathy levels in medical and dental students, as supported by research showing that empathy scores are significantly higher for students studying an integrated curriculum versus a traditional curriculum [13]. The complexity of conceptual integration in different courses is reflected by Harden's integration levels, which range from 1 to 10 [14]. The American University of Beirut Faculty of Medicine implemented a new curriculum in 2013-2014 to improve student well-being, enhance empathy, and counter the negative influences of the hidden curriculum. A longitudinal study in 2020 assessed the effectiveness of the new curriculum over seven years. Results showed higher learning environment and empathy scores among the cohorts following the new curriculum, although a decrease in empathy was observed in the third and fourth years. The new curriculum also improved students' perceptions and responses to the hidden curriculum [15].

Despite the established significance of integrated curriculum in increasing empathy scores, previous studies assessing the effect of differential educational strategies on empathetic behavior using various levels of integration are inadequate. In this study, we aimed to assess the effect of differential educational strategies on empathy scores using two different levels of integration. Level 7 (correlation-based teaching) involves incorporating integrated teaching sessions within a subject-based curriculum structure, promoting interdisciplinary understanding among students. Whereas, level 9 (multidisciplinary approach) integrates multiple subject areas into a single course or curriculum, focusing on themes, problems, topics, or issues [14-16]. The study aimed to determine whether differential levels of integration are associated with disparities in empathy scores and to measure the magnitude and direction of these relationships. This is crucial for recognizing whether a change in learning strategy induces changes in empathy scores. The study also aimed to identify whether the implemented approaches are independently correlated with empathy scores and to measure the magnitude and direction of these relationships.

Research objectives

(i) To assess if there is a difference in empathy scores based on the levels of integration, specifically between those instructed at Harden's integration level 7 (correlation-based teaching) and those at level 9 (multi-disciplinary teaching), among final-year medical and dental students at two institutions. (ii) To evaluate whether independent variables are associated with empathy scores in the medical and dental education curriculum at different levels of Harden’s integration ladder.

## Materials and methods

Subject recruitment

A quantitative research approach utilizing a cross-sectional study design was used to compare empathy scores at two different levels of integration on Harden’s ladder of curricular integration relevant to medical and dental education [14]. The study period for the research was eight months, from August 2023 to April 2024. Data were collected from the medical and dental institutions of Heavy Industries Taxila Education City-Institute of Medical Sciences (HITEC-IMS) in Taxila (Medical and Dental Colleges) and Shifa College of Medicine (SCM)/Shifa College of Dentistry (SCD) at Shifa Tameer-E-Millat University (STMU) in Islamabad. In these institutions, different curriculum integration approaches were regularly practiced; thus, there was no need to implement new interventions for increasing empathy scores. Therefore, the present study focused on analyzing the differences in the empathy scores of students as they relate to the differences in the implementation of these different curriculum approaches. Only final-year medical and dental students were included as two different samples from two different populations. The study included both male and female students who were taught at different curricular integration levels in medical and dental disciplines. Students who did not provide voluntary consent and those who were absent at the time of data collection were not included in the study.

A sample size of 256 students was estimated, as per calculations using G*Power (Heinrich-Heine-Universität Düsseldorf, Düsseldorf, Germany), which included 128 students from each program (Bachelor of Medicine and Bachelor of Surgery (MBBS) and Bachelor of Dental Surgery (BDS)) and 64 students from each independent group for generalization to larger populations. The effect size was set at 0.5 for sample size estimation purposes. A purposive sampling technique was used to collect the data. The sample comprised students taught at different curricular integration levels in medical and dental disciplines. Only final-year medical and dental students were recruited. 

In total, 300 students were approached; however, after refusals to participate in the study and discarding incomplete data, a final sample of 137 MBBS students and 73 BDS students was reached from the two institutions. The lower number of BDS students in the sample reflects the smaller dental student population at the two institutions compared to medical students. 

Data collection

The study utilized the Kiersma-Chen Empathy Scale-Revised (KCES-R) to assess empathy scores among participants (see Table 5 in Appendices) [17]. It was administered after permission was granted by the scale’s developer. The tool includes 14 items divided into two parts - Global Health Professional Empathy (GHPE) and Perceived Self-Empathy (PSE). Each part contains seven statements related to empathy. The tool was rated on a seven-point scale [17]. Student responses contributed to calculating their mean empathy score. A structured form was created to gather data on the sociodemographic characteristics of the participants. The Cronbach alpha value was 0.921, indicating that the scale had high reliability and that the findings for empathy measurement were reliable and consistent within the Pakistani context.

Students were approached after obtaining a no objection certificate (NOC) from the concerned heads of their respective colleges (Dean STMU and Principal/Dean HITEC-IMS). They were approached during their orientation sessions in wards with the assistance of the respective Heads of Departments or other authorized persons. Participants were verbally briefed about the research's significance and the value of their participation. Data were collected from participants who provided informed consent and met the inclusion criteria. After consent was obtained, responses were recorded on a questionnaire by the participants. Ultimately, around 300 students were approached, but only 210 responded, yielding a response rate of 70%. Data collection was completed in approximately one month. A p-value of less than 0.05 was considered statistically significant.

Data analysis

The IBM SPSS Statistics version 26 (IBM Corp., Armonk, USA) was used for data analysis. Before performing the analysis, the data were screened using normal Q-Q plots and boxplots to ensure that the necessary parametric assumptions were met, i.e., data were normally distributed, homogeneity of variance assumptions was satisfied, and variables were scored on continuous or interval scales and were independently observed. Descriptive statistics were conducted to analyze sociodemographic characteristics and the outcome variable. The data were summarized using frequencies and percentages, which were then presented in tabular format. Inferential analysis, including correlation and t-test analysis, was then performed to test the hypothesis.

Ethical considerations

Ethical approval was obtained from the Institutional Review Board (approval number 0296-23). Permission letters were received from the Dean of STMU and the principals of both HITEC-IMS Medical and Dental Colleges. The purpose of this research was explained to the respondents, and voluntary consent in written format was obtained from each participant before data collection. Only participants who voluntarily agreed to participate in the research process were included. Participants were assured of the confidentiality of their data, and all collected data was anonymized and kept confidential. Data were entered into IBM SPSS Statistics without identifying information. After data entry, hard copies of the collected data were secured in locked drawers and will be shredded after a year. 

## Results

Sociodemographic characteristics

A total of 210 students participated in the study. Among 137 medical students, 62.8% (n=86) were female participants, and 98% were within the age range of 22-25 years (n=135). Furthermore, among 73 BDS students, 82.2% (n=60) were female participants, and 95.9% were within the age range of 21-25 years (n=70). In the MBBS program, 51.8% (n=71), and in the BDS program, 54.8% (n=40), were from integration level 7. Detailed sociodemographic characteristics of the respondents are illustrated in Table 1.

**Table 1:** Sociodemographic characteristics of the respondents MBBS: Bachelor of Medicine and Bachelor of Surgery; BDS: Bachelor of Dental Surgery

Variable	Categories	MBBS (n=137)		BDS (n=73)		
		N	%	N	%	
Age	18-21 years	2	1.5	3	4.1	
	22-25 years	135	98.5	70	95.9	
Gender	Male	51	37.2	13	17.8	
	Female	86	62.8	60	82.2	
Curriculum integration level	Integration level 7	71	51.8	40	54.8	
	Integration level 9	66	48.2	33	45.2	

Statistical analysis

Pearson’s product-moment correlation analysis for the lower left MBBS students (Table 2) showed a significant relationship between GHPE, PSE, and the computed empathy score, as well as curriculum integration level. Here, GHPE was significantly positively correlated with the curriculum integration level. Moreover, PSE was positively significantly correlated with the curriculum integration level and GHPE. Furthermore, computed total empathy scores were positively correlated with the curriculum integration level, GHPE, and PSE.

**Table 2:** Relationship between curriculum integration levels and empathy scores with respect to MBBS and BDS program Lower left represents MBBS program and upper right represents BDS program. n: sample size; M: Mean; SD: Standard deviation; GHPE: Global Health Professional Empathy; PSE: Perceived Self-Empathy; MBBS: Bachelor of Medicine and Bachelor of Surgery; BDS: Bachelor of Dental Surgery; *: p<0.05 (one-tailed); **: p<0.01 (two-tailed)

Variable	n	M	SD	1.	2.	3.	4.
1. Curriculum integration level	137	0.47	0.50	-	0.28*	0.20	0.26*
2. GHPE	137	39.58	7.41	0.34**	-	0.59**	0.93**
3. PSE	137	37.80	6.95	0.33**	0.52**	-	0.90**
4. Computed empathy scores	137	77.39	12.94	0.38**	0.87**	0.87**	-

Pearson’s product-moment correlation analysis for the upper right BDS students (Table 2) also showed a significant relationship between GHPE, PSE, and the computed empathy score, as well as curriculum integration level. Again, GHPE was significantly positively correlated with the curriculum integration level. Additionally, PSE was positively significantly correlated with the curriculum integration level and GHPE. Furthermore, total computed empathy scores were positively correlated with curriculum integration level, GHPE, and PSE.

The results of the independent sample t-test (Table 3) showed a significant mean difference between integration level 9 and integration level 7, indicating that the empathy scores were higher at integration level 9 than at integration level 7. Additionally, there was a significant difference in GHPE and PSE scores across the two integration levels among MBBS students.

**Table 3:** Difference in empathy scores among MBBS students across curricular integration level 7 and integration level 9 P-values were calculated using the independent samples t-test. M: Mean; SD: Standard deviation; t: t statistic value;  P: Significance value; CI: Confidence interval, LL: Lower limit; UU: Upper limit; GHPE: Global Health Professional Empathy; PSE: Perceived Self-Empathy; MBBS: Bachelor of Medicine and Bachelor of Surgery

Variable	Integration level 7		Integration level 9		t	P	95% CI	
	M	SD	M	SD			LL	UL
GHPE	38.6	6.5	42.93	6.5	-4.28	0	-6.33	-2.33
PSE	36.94	6.08	41.09	5.69	-4.11	0	-6.13	-2.15
Computed empathy scores	75.55	10.4	84.03	9.79	-4.91	0.00	-11.8	-5.08

The results of the independent t-test (Table 4) further revealed that the difference between empathy scores at integration level 9 and integration level 7 was statistically significant, with empathy scores being higher at integration level 9 than at integration level 7. Additionally, there was a significant difference in GHPE scores across the two integration levels among BDS students.

**Table 4:** Difference in empathy scores among BDS students across curricular integration level 7 and integration level 9 P-values were calculated using the independent samples t-test. M: Mean; SD: Standard deviation; t: t statistic value;  P: Significance value; CI: Confidence interval, LL: Lower limit; UU: Upper limit; GHPE: Global Health Professional Empathy; PSE: Perceived Self-Empathy; BDS: Bachelor of Dental Surgery

Variable	Integration level 7		Integration level 9		t	P	95% CI	
	M	SD	M	SD			LL	UL
GHPE	35.27	9.51	40.21	7.21	-2.45	0.01	0.89	-0.92
PSE	34.25	7.86	37.36	7.31	-1.73	0.08	-6.68	0.46
Computed empathy scores	69.53	16.12	77.58	13.04	-2.35	0.02	-14.99	-1.1

## Discussion

The medical curriculum's patient-centric approach overlooks the humanitarian aspect of healthcare professionals, resulting in lower professional satisfaction and psychological well-being. Empathy-focused education is becoming increasingly popular in health professions education [7,18]. The term "integrated curriculum" has gained popularity in medical education over the past two decades, with many South Asian medical schools transitioning to problem-based learning curricula [19]. However, research on the impact of these curricula on professional skills like empathy and communication remains limited, despite most studies focusing on curriculum implementation. The present study aimed to find the differences in empathy scores of final-year medical and dental students trained to embody empathetic behavior at different levels of curricular integration as proposed by Harden.

The study found a significant positive association between empathy scores in the medical education curriculum and the integration levels of Harden's ladder. Higher levels of empathy were associated with more favorable educational experiences at higher integration levels. The study also found that level 9 instruction, which emphasizes multi-disciplinary teaching, was associated with higher levels of empathy in medical students compared to level 7 instruction, which uses correlation-based teaching [20]. This suggests that level 9 instruction is more effective in fostering empathy in medical students, highlighting the importance of integrating diverse teaching methods.

The study found a significant positive association between empathy scores in the dental education curriculum at different levels of Harden's integration ladder. It was observed that GHPE and total computed empathy were positively related to curriculum integration levels, but no significant relationship was found with PSE. Empathy has been linked to better patient quality of life, enhanced enablement, greater engagement, higher compliance, and less emotional distress [21]. However, insensitive treatment can irritate and disappoint patients, leading them to avoid medical professionals [22]. Empathy can help patients feel less afraid of dentists, develop better relationships with their doctors, cooperate more, comply more, and achieve better clinical results and higher patient satisfaction [22,23]. The findings suggest that the level 9 instructional approach promotes a greater understanding and appreciation of empathy in dental students.

The study also found significant differences in empathy scores between MBBS students with curricular integration at level 7 and level 9. This aligns with previous research showing that traditionally educated fifth-year students have higher empathy scores compared to those with student-centered curriculums. Additionally, students with an integrated modular curriculum who received formal ethics and professionalism training showed higher empathy levels [24].

Results revealed a statistically significant difference between empathy scores by levels of integration, with students taught at Harden's integration level 7 scoring lower than those at level 9 in final-year BDS at two institutions. The result of the independent t-test indicated that the empathy scores were higher at integration level 9 than at integration level 7 for BDS students.

The study found that empathy levels varied among students from different educational settings, with student-centered curriculums showing higher empathy scores than traditional ones. This finding is significant as it identifies differences in empathy scores across multiple health sciences disciplines. Medical students tend to have higher empathy levels than dental students [25], possibly due to longer and varied patient interactions during clinical rotations. In contrast, dental students may have more limited interactions, focusing on oral health and procedures rather than a holistic view of the patient's overall well-being [26,27].

The study highlights the need for targeted interventions and educational approaches to enhance empathy in medical and dental students. It emphasizes the importance of incorporating patient-centered care experiences and communication skills training into the curriculum. The study also outlines the unique challenges and opportunities for empathy development in different healthcare professions and the need for tailored approaches to cultivate empathy in future healthcare providers.

Limitations

The cross-sectional nature of the study made it difficult to identify cause-and-effect relationships, which can be addressed through a longitudinal study design. Additionally, generalizability for the dental students' sample was limited due to the unmet estimated sample size, which can be improved in future studies. Future research should also consider confounders such as individual differences, life experiences, and external factors.

## Conclusions

Medical and dental education needs to constantly adapt to meet the demands of the evolving healthcare landscape. One important aspect of medical and dental education is the development of empathy in future healthcare professionals. In conclusion, the findings of this study suggest a positive relationship between curricular integration and empathy scores in medical and dental students. By integrating empathy-focused curricula, educational institutions can better prepare students to provide patient-centered care and improve overall patient outcomes. Further exploration into the specific components of the level 9 curriculum that contribute to higher empathy scores will be invaluable for shaping the future of medical education.

## Appendices

**Table 5:** Kiersma-Chen Empathy Scale-Revised (KCES-R) version

KCES-R: The following questions pertain to your attitudes and feelings towards patients. Please mark the circle on the scale below that best represents your response.							
Part 1: How necessary is it for healthcare professionals to be able to…	Unnecessary	Moderately unnecessary	Somewhat necessary	Necessary	Somewhat necessary	Moderately necessary	Extremely necessary
Comprehend patient’s experiences.							
Express an understanding of patient’s feelings.							
Value patient’s point of view.							
Consider patient’s feelings to provide patient-centered care.							
Be caring in order to build a strong relationship with patient.							
Identify with patient’s feelings.							
View the world from patient’s perspective.							
Part 2: I am able to...	Doesn’t describe me at all	Doesn’t describe me very well	Doesn’t describe me well	Describes me slightly well	Describes me moderately well	Describes me well	Describes me very well
Comprehend patient’s experiences.							
Express an understanding of patient’s feelings.							
Value patient’s point of view.							
Consider patient’s feelings to provide patient-centered care.							
Be caring in order to build a strong relationship with patient.							
Identify with patient’s feelings.							
View the world from patient’s perspective.							
